# Does low alcohol use increase the risk of sickness absence? A discordant twin study

**DOI:** 10.1186/s12889-016-3502-2

**Published:** 2016-08-18

**Authors:** Kristian Amundsen Østby, Nikolai Czajkowski, Gun Peggy Knudsen, Eivind Ystrøm, Line C. Gjerde, Kenneth S. Kendler, Ragnhild E Ørstavik, Ted Reichborn-Kjennerud

**Affiliations:** 1 Department of Mental Health, Norwegian Institute of Public Health, Oslo, Norway; 2 Department of Psychology, University of Oslo, Oslo, Norway; 3 Virginia Institute for Psychiatric and Behavioral Genetics and Departments of Psychiatry and Human Genetics and Medical College of Virginia ⁄ Virginia Commonwealth University, Richmond, VA USA; 4 Department of Epidemiology, Columbia University, New York, NY USA

**Keywords:** Alcohol, Sick leave, Twin

## Abstract

**Background:**

Results from observational studies suggest that people who drink little or no alcohol are less healthy than medium drinkers. This has been demonstrated for many different measures of health, including sick leave. However, whether these associations are causal or due to confounding remains to be clarified. The aim of this study was to use a discordant twin design to determine whether the increased level of sick leave associated with a low level of alcohol consumption, as compared to those with a medium level of consumption, reflects a causal mechanism or is due to genetic or environmental confounding.

**Methods:**

Six thousand seven hundred thirty-four young adult twins from the Norwegian Institute of Public Health’s twin panel were in 1998 assessed for frequency of alcohol use and binge drinking. Data were linked to the Norwegian National Insurance Administration’s recordings of sick leave over a 10 year period. The associations between alcohol consumption and sick leave were first estimated in the total study population, and then within di- and monozygotic twin pairs discordant for alcohol use.

**Results:**

Compared to medium consumption, both low and high alcohol consumption was associated with increased risk of sick leave. When low level drinkers were compared to medium level drinkers in a discordant twin design, the results were consistent with the association being due to genetic confounding rather than a causal effect.

**Conclusions:**

The increased level of sick leave observed with low level drinkers seems to be mainly explained by confounding from genetic factors. In all observational studies of the relationship between alcohol consumption and health, one should be aware that important genetic confounders are likely to influence the results.

## Background

Damage from alcohol use is a serious health problem. Globally, alcohol use causes more deaths than e.g. HIV/AIDS, and is the leading risk factor for death in males aged 15–59 [1]. In middle-income countries, alcohol is the single greatest risk factor for disease and disability, and WHO estimates a net loss of 2.25 million lives each year due to alcohol related causes [1].

Moderate alcohol consumption, on the other hand, is often presented as possibly health-promoting, and has been proposed to protect against several diseases [2–4]. This assumption is based on an observed curve-linear relationship between alcohol consumption and various health measures, where both low and high level drinkers seem to have an increased risk when compared to moderate drinkers. Some believe that the dis-favourable outcome among low level drinkers is due to a genuine health-promoting effect of alcohol [5]. Others argue that these findings are more likely to be caused by unmeasured confounding (i.e. genetic or environmental factors influencing both alcohol exposure and the outcome) [6–9], and in some cases the effect has been explained by inclusion of “sick quitters” (people who quit drinking due to medication, health problems or even alcohol problems) in the abstainer group [10, 11].

In observational studies, participants should ideally differ from each other only in terms of exposure. If test groups differ on other aspects there is a risk of confounding, meaning that a third factor influencing both exposure and outcome creates a spurious relationship between the two that may be misinterpreted as causal. Many confounders are difficult to measure or adjust for properly, and unmeasured confounders pose an important challenge.

One way to reduce the problem of unmeasured confounding is to apply a *discordant twin design* [12], a method designed to eliminate confounding by familial factors (early shared environment and/or common genetic factors). Twins reared together share early childhood environment and monozygotic (MZ) twins are genetically identical while dizygotic (DZ) twins share on average 50 % of their genes. In a discordant twin study the difference in outcome within twin pairs that are *discordant* for the exposure variable (i.e. only one of the twins is exposed for the risk factor) is examined. If e.g. low use of alcohol has a causal negative effect on health, one would expect a twin with medium alcohol consumption to be of better health than his or her abstaining twin. If, on the other hand, the association between alcohol consumption and health is fully confounded by childhood environment or genetic factors, no differences in health would be expected. A discordant twin design will also help distinguishing between environmental and genetic confounding by comparing the results for MZ and DZ twin pairs.

Discordant twin studies have previously been conducted on the relationship between alcohol use and outcomes like type 2 diabetes [13] and bone mineral density [14]. But to the best of our knowledge, no such study has yet focused on the relationship between alcohol consumption and sick leave, which could be seen as a more general indicator of health [15, 16]. Sick leave is known to be related to alcohol consumption [17–20] and both abstainers and high level users have been found to be at increased risk when compared to moderate users [21, 22]. Thus, the associations between alcohol consumption and sick leave seem to follow the same curve-linear relationship as that of other health measures. However, in none of these previous studies of alcohol use and sick leave has it been possible to adjust for unmeasured familial factors. It is therefore unknown whether the increased levels of sick leave associated with low level drinking reflects a causal relationship or is due to confounding.

We wanted to address this question using a discordant twin design. In 1998 a population-based sample of Norwegian twins was assessed for alcohol consumption, and we have linked this data to sick leave data from official Norwegian welfare registries for the period from 1998 to 2008. In the current paper we first explore the relationship between sick leave and alcohol use, estimating the differences in sick leave between those with a low level and those with a medium level of consumption. Then we use a discordant twin design to investigate whether any differences in sick leave between the two groups reflect a causal relationship or can be explained by confounding by childhood environment and/or genetic factors.

## Methods

### Study sample

The Norwegian Institute of Public Health Twin Panel includes information on all twins born in Norway between 1967 and 1979 [23]. The twins are identified through the Norwegian Medical Birth Registry, which receives mandatory notifications of all live- and stillbirths of at least 16 weeks of gestation. In 1998, all complete twin pairs, a total of 12,698 twins, were invited to take part in a questionnaire based study on various mental and somatic symptoms and disorders, including alcohol use. In this study 8045 twins (3334 complete twin pairs and 1377 single twins) participated, giving a response rate of 63 % [24]. Using personal identification numbers issued to all Norwegians at time of birth, the questionnaire data was linked to the Norwegian National Insurance Administration’s records from 1998 to 2008. A total of 335 participants withdrew from the study before this linking was carried out.

The welfare registries are updated annually and their accuracies are well documented [25]. They record all episodes of sick leave exceeding 16 days, as well as employment status. Normally, only periods of sick leave beyond 16 days are covered by public welfare program, and shorter periods of sick leave are therefore not included in the official registries.

### Measures

#### Exposure: Alcohol use

We utilized two measures of alcohol use; frequency of use and binge drinking. Frequency of alcohol use was derived from the question “How often did you drink alcohol (beer, wine or liquor) during the last 14 days?” Response options were “I am an abstainer, I never drink alcohol”, “I did not drink alcohol but I am not an abstainer”, “1–4 times”, “5–10 times” and “More than 10 times”. Such a measure of alcohol consumption is commonly used in research, and has been shown to be a predictor of both future work participation [10, 22] and future health [10, 26]. Respondents who confirmed to be an abstainer or had not been drinking alcohol during the past 14 days were classified as low level users.

The binge drinking variable was derived from the question: “How often do you drink an amount of alcohol corresponding to five or more [alcohol units]?” (referred to as “binge drinking” throughout this paper). Response options were “0–4 times a year”, “5–10 times a year”, “1–3 times a month”, “1–2 times a week”, “3–5 times a week” or “6 times or more a week”. Participants who had confirmed to be an abstainer, and participants who reported 0–4 episodes of binge drinking for the past year, were classified as low level users.

Heavy drinking poses a well-known health risk [27], and is also known to be associated with increased levels of sick leave [18]. Because we were interested in the possible health risks associated with low level use, we excluded from the discordant twin analyses those with the highest levels of alcohol use. For the analyses of frequency this meant those with more than ten drinking episodes during the past 14 days. For the analyses of binge drinking those who reported three or more weekly episodes of binge drinking throughout the past year were excluded. These participants are referred to as high level users throughout this paper.

For the analyses of frequency of use, the reference group (referred to as medium level users) consequently comprised responders who had been drinking from 1 to 10 times during the past 14 days. For the analyses of binge drinking, the reference group comprised participants reporting binge drinking between 5 times per year and 2 times per week.

#### Outcome: Sick leave

In Norway, a continuous period of sick leave is limited to 52 weeks. After that an individual who is still unable to work must usually undergo medical/vocational rehabilitation, a period of treatment or training aimed at regaining work ability, before eventually returning to work or qualifying for a disability pension. For the period of 1998–2008 we counted all days of either sick leave or rehabilitation grants as *sick days*. Days with a recorded employment was counted as *expected work days*. For participants with at least 100 expected work days during the 11 year follow up period, we defined a continuous sick leave variable based on their number of *sick days* divided by their *expected work days*. This variable was used as the outcome variable, and is referred to as *sick leave* in the remainder of this article.

### Statistical analyses

In Norway alcohol use is uncommon during pregnancy, and pregnancy is a well-documented risk factor for sick leave [28]. We therefore excluded women who had given birth in 1998 or 1999 (825 participants) since they were likely to have been pregnant at the time alcohol consumption was assessed. We also excluded an additional 109 participants who had less than 100 work days registered throughout the ten year follow up period, and 42 participants who had missing data on both alcohol variables.

To be able to conduct comparisons within opposite-sex dizygotic pairs, we adjusted for sex differences in sick leave by first estimating the alcohol adjusted difference in sick leave rates between men and women in the total study population. This difference was then subtracted from every woman’s sick leave. Women tend to drink less, and their low level of alcohol use might theoretically be the cause of their increased sick leave. It was therefore important to adjust the sick leave for alcohol adjusted sex differences. Had we instead adjusted merely for sex differences in sick leave, we might have removed any possible effects on women’s sick leave caused by low use of alcohol.

Discordant twin analyses were carried out twice, once for each measure of alcohol use, and in both analyses the predictor variable was treated as a dichotomous variable, comparing low level users to medium level users. High level users were omitted from the analyses as mentioned above. The association between the dichotomous measures of alcohol use and sick leave was first estimated in the total sample using linear regression analyses, and the dependency within twin pairs was corrected for by using generalized estimating equations (GEE) with exchangeable covariance structure [29]. The association was then tested within twin pairs discordant for alcohol use, using paired sample t-tests. This was done separately for MZ and DZ twin pairs. All these statistical analyses were conducted in SPSS version 17.0 for Windows. Finally we tested for significant differences in the estimates across the groups (DZ pairs, MZ pairs and the total population) using the following formula:

$$ \mathrm{t} = \left|{\mathrm{e}}_1\hbox{-} {\mathrm{e}}_2\right|/\surd \left({{\mathrm{SD}}_1}^2/{\mathrm{n}}_1+{{\mathrm{SD}}_2}^2/{\mathrm{n}}_2\right)\ {\mathrm{e}}_{\mathrm{x}} = \mathrm{estimate}\ \mathrm{in}\ \mathrm{group}\ \mathrm{x},\ {\mathrm{SD}}_{\mathrm{x}} = \mathrm{standard}\ \mathrm{deviation}\ \mathrm{in}\ \mathrm{group}\ \mathrm{x} $$

**Fig. 1:**
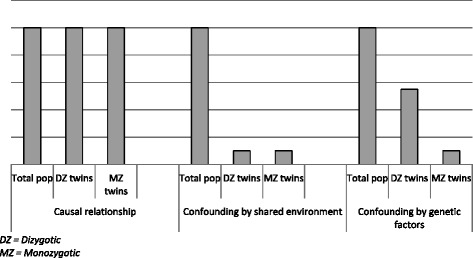
Prototypical risk patterns found in discordant twin analyses

Depending on the nature of the association, one of three main patterns can be expected to be found in a discordant twin analysis. If the relationship between exposure and outcome is causal, and not confounded by childhood environment or genetic factors, the risk will be the same in the total population as within discordant twin pairs (*left model in* Fig. 1). If the relationship is non-causal and *confounded by early shared environment*, there will be a lowered risk within discordant twin pairs than in the total population, and the risk will be the same within di-zygotic pairs as within mono-zygotic pairs (*middle model in* Fig. 1). If the relationship is non-causal and *confounded by genetic factors*, the risk will be lower within mono-zygotic pairs than within di-zygotic pairs (*right model in* Fig. 1).

## Results

**Fig. 2:**
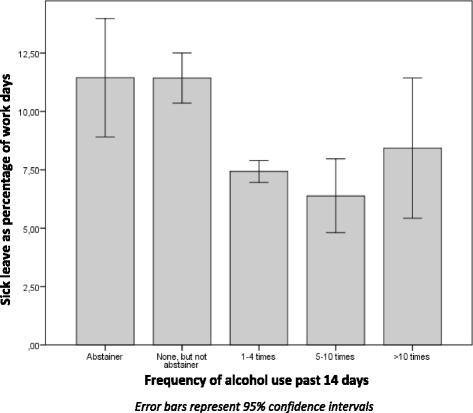
Frequency of alcohol use and sick leave in total population

**Fig. 3:**
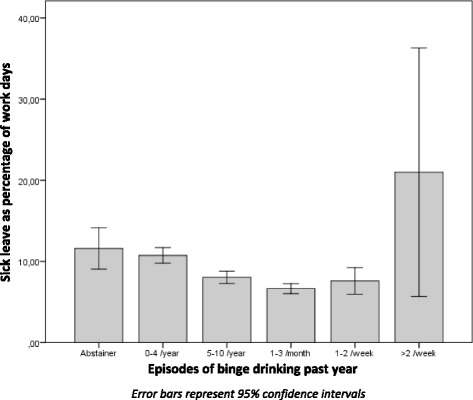
Binge drinking and sick leave in total population

The final sample consisted of 6734 participants, 19–31 years of age (mean 25.3), of which 52.6 % were women (Table 1). The relationship between alcohol use and sick leave is shown in Figs. 2 and 3. Both for frequency of alcohol use and for binge drinking there was a non-linear relationship with sick leave. There were no significant differences between dizygotic and monozygotic twins in alcohol use or in level of sick leave.

**Table 1:** Study sample

	Female sex	Mean age at baseline (SD^a^)	Sick leave (SD^a^)
Total population (*n* = 6734)	52.6 %	25.3 (3.7)	8.5 % (17.9)
Alcohol use past 14 days			
No use (*n* = 1911)	59.1 %	25.3 (3.8)	11.4 % (22.0)
1–10 times^b^ (*n* = 4714)	50.1 %	25.3 (3.7)	7.3 % (15.8)
> 10 times (*n* = 105)	43.8 %	24.9 (3.7)	8.4 % (15.5)
Binge drinking past year			
0–4/year (*n* = 2053)	63.2 %	26.0 (3.8)	10.9 % (20.9)
5/year - 2/week^b^ (*n* = 4562)	47,6 %	25.0 (3.7)	7.3 % (15.9)
> 2/week (*n* = 18)	16.7 %	25.2 (3.5)	21.0 % (30.8)

^a^Standard deviation

^b^Reference group

As can be seen from Fig. 2, the low level users (abstainers and those who had not been drinking for the past 14 days, *n* = 1911) had higher levels of sick leave than the medium level users (those reporting 1–10 times of alcohol use, *n* = 4714). The high level users (reporting >10 times of use, *n* = 105) also had increased levels of sick leave.

Figure 3 shows similar results for binge drinking: The low level users (the abstainers and those reporting 0–4 episodes of binge drinking during the last year, *n* = 2053) had increased levels of sick leave when compared to the medium level users (reporting between five episodes of binge drinking per year and up to 2 times per week, *n* = 4562). The high level users (reporting 3 or more weekly episodes of binge drinking for the past year, *n* = 18) also had increased levels of sick leave.

**Fig. 4:**
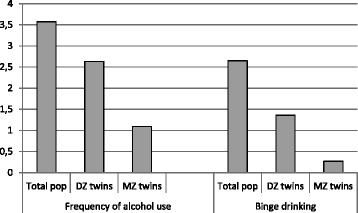
Results from discordant twin analyses of alcohol use and sick leave

For both alcohol measures the differences in sick leave between low level and medium level users are shown in Fig. 4, first within the total population, then within DZ and MZ twin pairs. Both these patterns mostly resemble the rightmost risk pattern in Fig. 1, indicating that the increased sick leave associated with low use of alcohol is not due to a causal effect of alcohol, but can rather be explained by confounding by genetic factors.

For frequency of alcohol use, the sick leave in the low level user group was on average 3.57 percentage points higher than that in the medium level user group (Table 2). Differences within twin pairs that were discordant for low or medium level use were 2.63 for DZ pairs and 1.09 for MZ pairs, with the difference within MZ pairs not being significant. The estimate for MZ pairs was also significantly different from the estimate in the total population, both before (*p* = 0.016) and after (*p* = 0.045) adjusting for sex differences in the sick leave. This pattern suggests confounding by genetic factors.

**Table 2:** Discordant twin analyses. Difference in sick leave between low and medium level users of alcohol^a^

	Total population (*n* = 6734)	Total population adjusted for sex	Dizygotic pairs adjusted for sex	Monozygotic pairs
Frequency of alcohol use	4.09 (2.94–5.25)	3.57 (2.42–4.71)	2.63 (0.36–4.90) (*n* = 434 pairs)	1.09 (−1.05–3.23) (*n* = 222 pairs)
Binge drinking	3.57 (2.48–4.66)	2.65 (1.58–3.72)	1.36 (−0.94–3.65) (*n* = 422 pairs)	0.27 (−2.21–2.75) (*n* = 181 pairs)

^a^Difference measured as low level user’s sick leave minus medium user’s sick leave. 95 % confidence intervals are shown in parentheses

For binge drinking the difference between low level users and medium level users was 2.65 in the total population, 1.36 within DZ pairs and 0.27 within MZ pairs (Table 2). Even though this pattern also mostly resembles that of genetic confounding, the differences were non-significant both within DZ and MZ pairs and therefore confounding by shared environment cannot be ruled out. The estimate for MZ pairs was also only significantly different from the estimate in the total population before adjusting for sex differences in sick leave (*p* = 0.017).

## Discussion

Our results confirmed the non-linear relationship between alcohol use and sick leave that is known from previous studies: A pattern with increased levels of sick leave for those with the lowest and highest levels of alcohol consumption was present both for frequency of alcohol use and for binge drinking. Even quite frequent binge drinking (up to 2 times per week) was associated with a lower level of sick leave when compared to never or rarely binging (0–4 times per year).

Associations like these, controlled for measured confounders, have sometimes been interpreted as a support for the claim that moderate alcohol consumption has a positive effect on health [3]. However, when we used discordant twin analyses we did not find that low alcohol consumption was associated with increased sick leave. This applied both to frequency of use and to binge drinking. Moderate alcohol consumption, as compared to low or no consumption, therefore does not seem to have a causal protective effect on sick leave.

Differences in sick leave were also lower within discordant MZ pairs than within discordant DZ pairs, indicating that the confounding is likely to be mainly caused by genetic rather than shared environmental factors. Our study does not reveal which specific genetic factors are involved, but they must be associated with alcohol use and also influence sick leave. One possibility is genetically inherited illnesses that lead to a low alcohol consumption. If alcohol consumption is reduced due to illness or medication, one would expect to see a positive association between low alcohol consumption and sick leave in the total population. But if two MZ twins, both having the same condition, are discordant for alcohol consumption, there would be no difference in sick leave between them if the sick leave is caused by the illness rather than by the difference in alcohol consumption. Another possible source of confounding is genetic factors influencing personality traits. Such traits have repeatedly shown to be heritable [30]. Several studies have shown that people with low alcohol consumption are less outgoing and score lower on extroversion than moderate drinkers [31, 32]. Personality is also known to be linked to other health behaviour [33–35] and to work participation in general [36]. If low drinkers have different personality traits than moderate drinkers, genetic factors influencing personality could work as a confounder. This is seldom adjusted for in observational studies of alcohol use and health.

Even though our findings indicate that genetic confounding is important, several environmental confounders could still be relevant. Discordant twin studies do not adjust for environmental factors that are not shared between the twins. As an example, we excluded pregnant women from our study. Pregnant women commonly have a low alcohol consumption and high risk of sick leave, and including them would have increased the association between low alcohol consumption and sick leave. Such confounding could not have been revealed by discordant twin analyses. This shows the importance of taking possible non-genetic confounders into account, even in genetic studies, and it should be a reminder that there are likely many unmeasured environmental confounders that may cause spurious associations in observational studies.

### Strengths and limitations

This study has several strengths. First of all, it is a genetically informed population based study. In addition, it utilizes accurate and objective data from official Norwegian registries on sick leave. Our results must still be interpreted in light of some limitations. Firstly, this was a study of young adult Norwegian twins. For the study of both mental and somatic health, twins have been shown to be representative for the general population [37, 38], but our results may not apply to other age- and ethnic groups. In a young population, somatic illness due to high alcohol consumption is not very common, but we still found an initial strong association between low alcohol use and sick leave in our sample. This strengthens our beliefs in the finding that the observed associations are not likely causal, but rather seem to be explained by confounding from genetic factors. In an older population alcohol damages might be more prominent, as would any protective effects. However, if the association between low alcohol consumption and sick leave in a young age group is explained by genetic factors, for example by personality, this confounding might also be relevant in older age groups. Thus, one should be aware of the possible presence of important genetic confounders in observational studies of alcohol use in any age group.

Secondly, abstainers and people with high alcohol consumption are less likely to participate in population based health studies [39]. Such participation bias is likely more problematic for studies of prevalence than studies like ours which focus on associations [40]. In our study it may have reduced the statistical power, as discordant twin analyses require a high number of participants. Even though some of our confidence intervals were wide, there was a clear trend in the estimates, indicating confounding by genetic factors as we have described.

Third, self-reported alcohol use might be an unreliable measure. We therefore think it is an advantage of this study that we use two different measures of alcohol use. We do not believe there are actual health benefits from binge drinking, but if confounding factors are influencing the relationship between regular alcohol use and sick leave, the same confounding factors could also be influencing the relationship between binge drinking and sick leave. The fact that we found similar patterns of confounding for two different measures of alcohol use therefore strengthens the credibility of our findings.

Fourth, even though a discordant twin design eliminates genetic and shared environmental confounding, it does not eliminate confounding by environmental factors that are not shared within twin pairs [41]. One example is that of pregnant women which, if they had been included in our study, would have influenced our results by increasing the association between low alcohol use and increased sick leave. There are many other possible environmental confounders that were not covered by this study, for example education and type of occupation. However, in our data, nearly the entire association between alcohol use and sick leave could be explained by familial confounding, and the inclusion of additional environmental factors in the model was therefore not strictly necessary.

Fifth, the registries that this study is based on do not record periods of short term sick leave totalling less than 16 days. Heavy alcohol use and binge drinking in particular are known to be associated with increased short term sick leave, likely due to “hangovers” [18, 42]. However, such acute short-term effects of intoxication are not very relevant when studying whether moderate alcohol consumption (as compared to low level use) has a positive effect on health in general. Long-term sick leave is therefore likely to be a better indicator of general health. And since sick leave exceeding 15 days comprises about two-thirds of the total number of sick days in Norway [43], long term sick leave is also a more important outcome in regard to societal costs.

## Conclusions

The increased level of sick leave observed with low level drinkers – and consequently the apparent protective effect of moderate alcohol consumption – seem to be mainly explained by confounding from genetic factors. These factors – whatever they are – might also be important for the relationship between alcohol use and other health measures. Consequently, in all observational studies of the relationship between alcohol consumption and health, one should be aware that important genetic confounders are likely to influence the results.

## Abbreviations

CI: Confidence interval
DZ: Dizygotic
MZ: Monozygotic
SD: Standard deviation
